# Integrative ChIP-seq/Microarray Analysis Identifies a CTNNB1 Target Signature Enriched in Intestinal Stem Cells and Colon Cancer

**DOI:** 10.1371/journal.pone.0092317

**Published:** 2014-03-20

**Authors:** Kazuhide Watanabe, Jacob Biesinger, Michael L. Salmans, Brian S. Roberts, William T. Arthur, Michele Cleary, Bogi Andersen, Xiaohui Xie, Xing Dai

**Affiliations:** 1 Department of Biological Chemistry, School of Medicine, University of California Irvine, Irvine, California, United States of America; 2 Institute for Genomics and Bioinformatics, University of California Irvine, Irvine, California, United States of America; 3 Department of Computer Science, University of California Irvine, Irvine, California, United States of America; 4 Rosetta Inpharmatics, LLC, Merck & Co Inc., Seattle, Washington, United States of America; Casey Eye Institute, United States of America

## Abstract

**Background:**

Deregulation of canonical Wnt/CTNNB1 (beta-catenin) pathway is one of the earliest events in the pathogenesis of colon cancer. Mutations in *APC* or *CTNNB1* are highly frequent in colon cancer and cause aberrant stabilization of CTNNB1, which activates the transcription of Wnt target genes by binding to chromatin via the TCF/LEF transcription factors. Here we report an integrative analysis of genome-wide chromatin occupancy of CTNNB1 by chromatin immunoprecipitation coupled with high-throughput sequencing (ChIP-seq) and gene expression profiling by microarray analysis upon RNAi-mediated knockdown of CTNNB1 in colon cancer cells.

**Results:**

We observed 3629 CTNNB1 binding peaks across the genome and a significant correlation between CTNNB1 binding and knockdown-induced gene expression change. Our integrative analysis led to the discovery of a direct Wnt target signature composed of 162 genes. Gene ontology analysis of this signature revealed a significant enrichment of Wnt pathway genes, suggesting multiple feedback regulations of the pathway. We provide evidence that this gene signature partially overlaps with the Lgr5^+^ intestinal stem cell signature, and is significantly enriched in normal intestinal stem cells as well as in clinical colorectal cancer samples. Interestingly, while the expression of the CTNNB1 target gene set does not correlate with survival, elevated expression of negative feedback regulators within the signature predicts better prognosis.

**Conclusion:**

Our data provide a genome-wide view of chromatin occupancy and gene regulation of Wnt/CTNNB1 signaling in colon cancer cells.

## Background

Wnt/CTNNB1 signaling is a conserved pathway that plays fundamental roles in embryonic development, tissue homeostasis and maintenance of stem cells. In normal intestine, this pathway is essential for the development and maintenance of intestinal stem cells [1], [2]. Activation of canonical Wnt signaling involves stabilization of cytoplasmic CTNNB1, which is otherwise degraded by the proteasome through a degradation complex composed of tumor suppressor protein APC, serine/threonine kinase GSK-3, and Axin. Stabilized CTNNB1 translocates into the nucleus where it binds to TCF/LEF transcription factors and activates target gene expression [2]. A key initiation event of colorectal cancers (CRCs) is CTNNB1 stabilization through loss of the *APC* gene or activating mutations in the *CTNNB1* gene [3]. This genetic event occurs primarily in the intestinal stem cell population [4]–[6] and leads to abnormal proliferation of mutated cells through activation of target genes such as *MYC* and *CCND1* [7]. However, given the divergent cellular roles of Wnt/CTNNB1 signaling, other target genes may also contribute to the pathogenesis of CRCs.

Thus, identification and characterization of CTNNB1 target genes genome-wide have been an important pursuit in CRC studies. Transcriptional profiling was used to identify Wnt/CTNNB1 target genes in colon cancer cells [8]. However, analysis of gene expression alone is limited due to inability to distinguish between primary and secondary effects of Wnt pathway activation. More recently, chromatin immunoprecipitation (ChIP) followed by large-scale DNA analysis such as DNA-chip (ChIP-chip) or high-throughput sequencing (ChIP-seq) [9], [10] was used to identify the genomic loci to which CTNNB1 or TCF factors directly bind [11]–[14]. However, ChIP-based studies of various transcription factors suggest that not all binding events identified correlate with transcriptional regulation at a functional level [15]. Therefore, it is important to perform an integrative analysis incorporating both genome-wide chromatin occupancy mapping and gene expression profiling in the same type of colon cancer cells in order to identify direct and functional Wnt/CTNNB1 target genes, which has not yet been done in the literature.

In the current study, we combine ChIP-seq with microarray analysis using the SW480 human colon cancer cell line, where deregulated Wnt signaling activity has been well documented. We report a significant correlation between CTNNB1 binding and expression regulation, and the identification of a core set of 162 genes as ‘Wnt direct target genes’ that are bound and activated by CTNNB1. The Wnt target gene signature was enriched in normal intestinal stem cells and CRCs but failed to predict prognosis of CRC patients. Our study provides an important resource for understanding the biology of Wnt signaling.

## Results

### ChIP-seq analysis of CTNNB1 chromatin occupancy in SW480 cells

**Figure 1:**
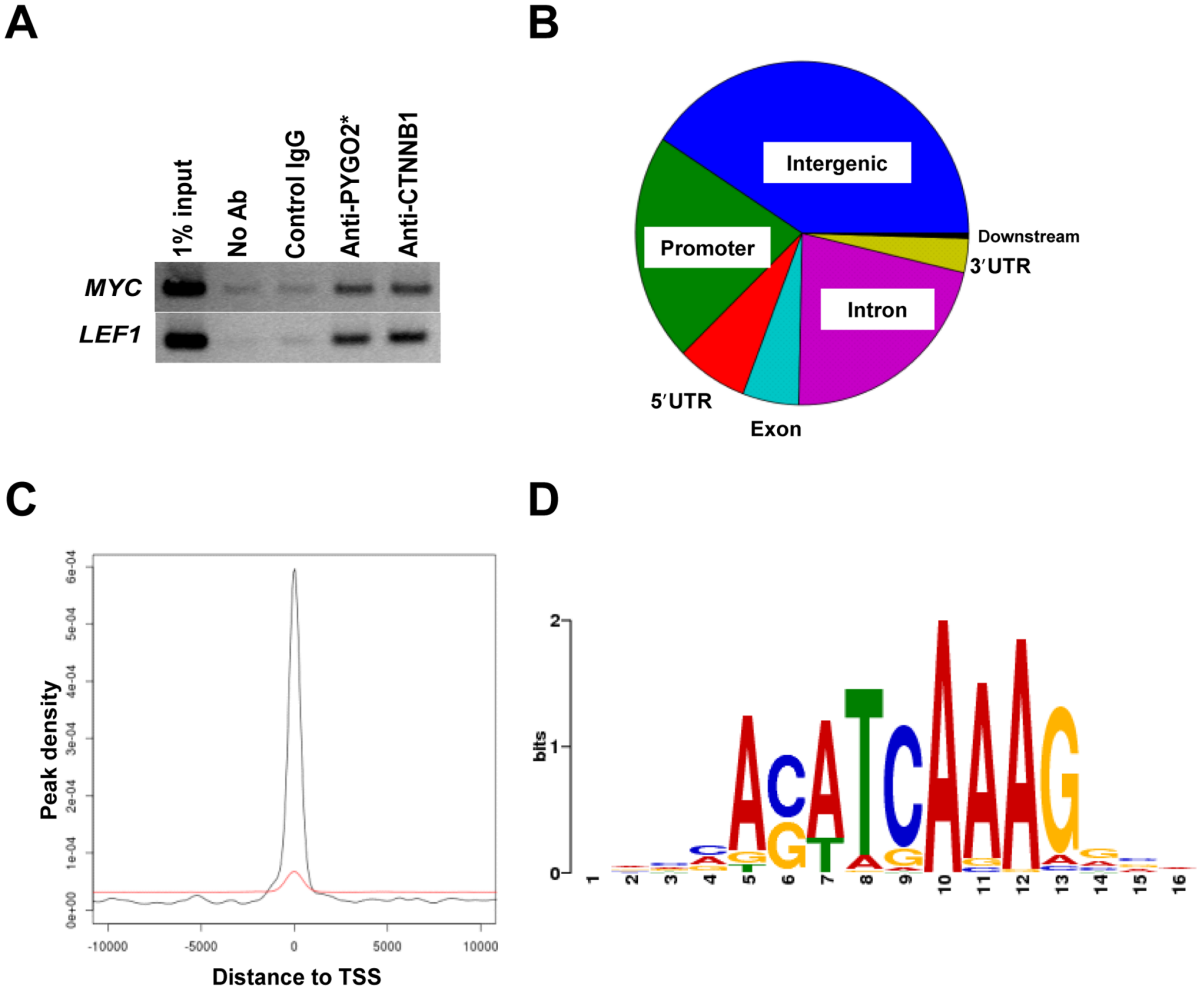
Peak distribution and motif analysis of CTNNB1 ChIP-seq. *A.* ChIP optimization. ChIP-PCR was performed for known CTNNB1-binding elements in MYC and LEF1 gene regulatory regions. *anti-PYGO2 antibody was used as a positive control, as PYGO2 protein associates with the CTNNB1-TCF/LEF protein complex [51]. *B.* Distribution of CTNNB1-binding regions on the genome relative to RefSeq human genes. A ‘promoter’ is defined as the 2-kb region upstream of the TSS, whereas a ‘downstream’ region is defined as the 2-kb region downstream of the transcription termination site. Intergenic region refers to all locations other than ‘promoter’, 5′-UTR, ‘exon’, ‘intron’, ‘3′-UTR’, or ‘downstream’. *C.* Distance from the center of each CTNNB1 binding peak to the TSS of the nearest gene. The red line represents a randomly distributed pattern generated by taking 1000 random permutations of the peaks. *D.* MEME program identifies a consensus TCF/LEF-binding motif within the ChIP-seq peaks.

SW480 cells contain an inactivating mutation in the *APC* gene that results in a high level of constitutively stabilized CTNNB1 protein in the nuclei [16], [17]. To capture the presumably dynamic interaction of CTNNB1 with chromatin [18], [19], we optimized a ChIP protocol using amine-specific protein-protein cross-linkers prior to formaldehyde cross-linking coupled with nuclear extraction [20] (See Methods). Specific binding of CTNNB1 was first validated by ChIP-PCR for known targets including *MYC* and *LEF1* (Figure 1A). DNAs from multiple ChIP experiments using anti-CTNNB1 antibody or isotype control IgG control were pooled for Solexa/Illumina sequencing, which yielded approximately 26 million individual 36-nucleotide sequence reads in each run. Only reads uniquely mapped on the human genome (15,776,628 for control IgG and 17,112,552 for anti-CTNNB1) were used for the subsequent analysis (Table 1). Model-Based Analysis of ChIP-Seq (MACS) [21] identified 3629 CTNNB1 binding peak regions (*p* value ≤ 1E-5, FDR ≤ 5%) across the entire genome (Table 1). Approximately 60% of the peaks are located in a close proximity of gene-encoding regions including promoter (2-kb 5′ flanking region, 21.5%), 5′-UTR (6.9%), exon (5.5%), intron (21.6%), 3′-UTR (3.2%) and downstream (2-kb 3′ flanking region, 0.5%) regions (Figure 1B). Enrichment of CTNNB1 binding at the promoters (21.5%) was statistically significant (*p*<0.001) when compared to the distribution of randomly generated peaks of similar sizes (3.5±0.2%). Consistent with a previous report [12], peak distribution correlated significantly with the proximity of the transcriptional start sites (TSS) (Figure 1C). Since a CTNNB1/TCF-bound enhancer can act remotely from the TSS [18], we sought for TSSs that are located within 20 kb from the observed peaks, and identified 2794 genes that were used in the subsequent analysis to identify the direct target genes.

**Table 1:** Summary of MACS analysis of the ChIP-seq data.

	Uniquely mapped reads	Peaks identified (FDR <5%)	Genes identified (< 20 kb)
CTNNB1	17,112,552	3,629	2794
IgG	15,776,628	/	/

Using Multiple EM for Motif Elicitation (MEME), a de novo motif discovery program [22], we analyzed the unique CTNNB1 peaks for the presence of any possible consensus motif. An extended TCF/LEF consensus sequence [12], [13] surfaced as the top ranked motif (E value  =  7.9e-1135) (Figure 1D). Several other candidate sequences were also identified; however, they seemed to represent repetitive sequences, which are common artifacts of this analysis (data not shown). These data confirm that CTNNB1 binds to the chromatin primarily through TCF/LEF DNA-binding proteins [11]. This said, we cannot exclude the potential binding motifs that have not been detected by MEME, as well as the existence of other associated motifs that lie outside of the ChIP peak regions [12], [14]. Indeed, a k-mer search [23] revealed that the AP-1 consensus sequence (TGAYTCA) is significantly enriched compared to similarly sized random genomic fragments (*p*  =  1.78e-50), consistent with previous work reporting the enrichment of AP-1 motifs in CTNNB1-binding elements [12].

**Figure 2:**
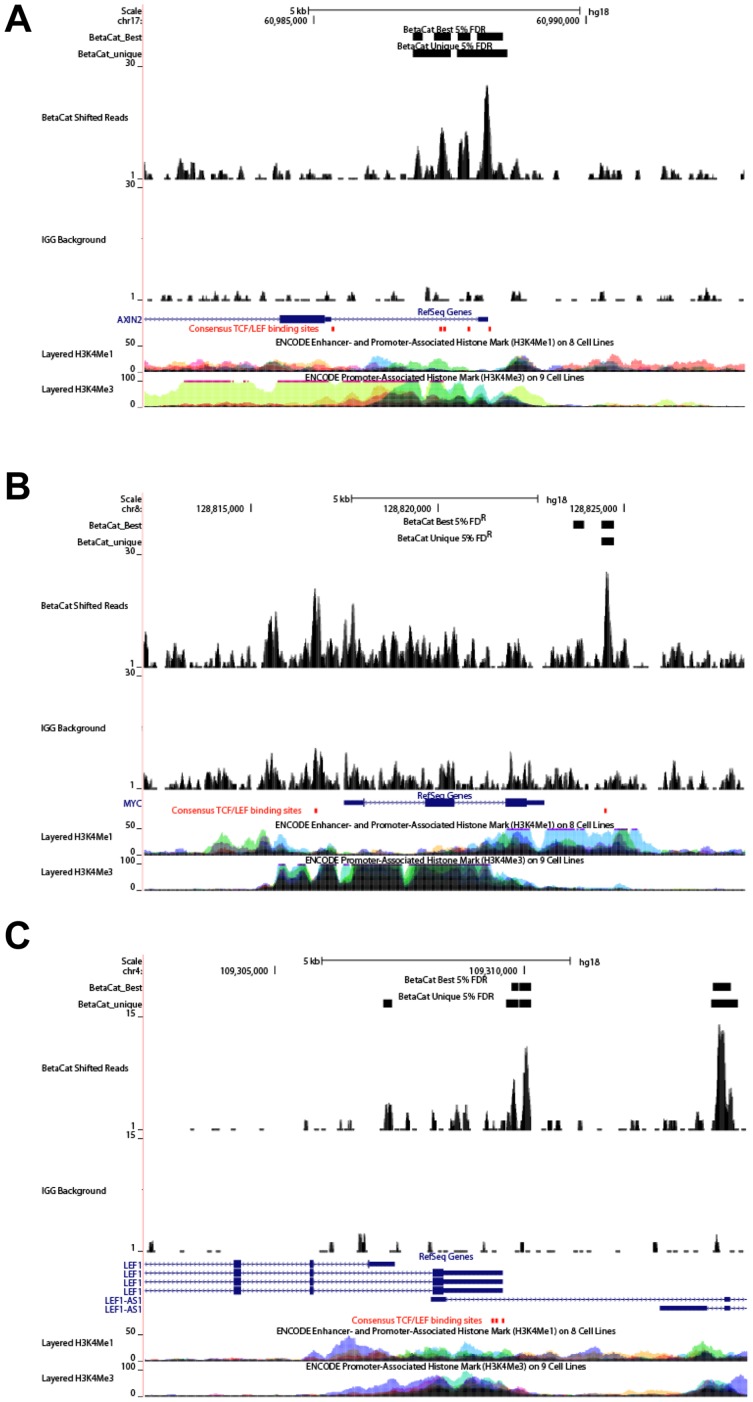
Visualization of the CTNNB1 binding peaks in *AXIN2* (*A*), *MYC* (*B*), and *LEF1* (*C*) genes. Shown are results for CTNNB1 and IgG distributions on Ref-seq gens. Red boxes indicate the positions of consensus TCF/LEF-binding motifs. H3K4me1 and H3K4me3 domains from multiple Encyclopedia of DNA Elements (ENCODE) cell lines are shown as references for enhancer/promoter and promoter regions, respectively.

Using the UCSC Genome browser (NCBI36/hg18) [24], we visualized CTNNB1 binding to known Wnt/CTNNB1 target genes. *Axin2* is a classical target and contains multiple TCF/LEF binding regions in its promoter and first intron [25]. Our ChIP-seq analysis indeed identified multiple peaks in *AXIN2*, with their positions corresponding well with the reported binding sites (Figure 2A). We also detected peaks at the reported regions of other known target genes including *MYC* [12] and *LEF1* [26] (Figure 2B and C).

### Combinational analysis of chromatin binding and gene expression data identifies a direct CTNNB1 target gene signature containing feedback regulators of the Wnt signaling pathway

**Figure 3:**
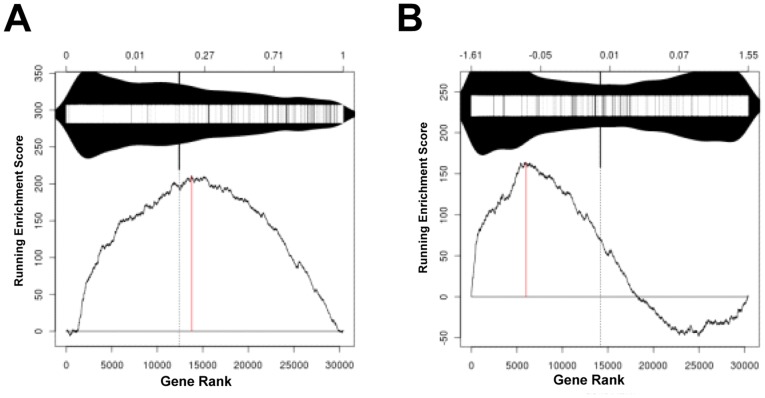
Comparative analysis of ChIP-seq and microarray data. *A-B*. KS plots showing correlation between CTNNB1 binding and expression regulation. The 2794 CTNNB1-bound genes were compared for their correlation with expression changes upon siRNA knockdown of CTNNB1. Genes in the microarray were ranked by *p* value (*A*, *p*  =  4.4e-16) or fold increase (*B*, *p*  =  0) in each GSEA.

To identify direct and functionally relevant CTNNB1 target genes, we analyzed the ChIP-seq dataset against transcriptional profiling data of control and CTNNB1-depleted SW480 cells [27]. As previously described [27], knockdown of CTNNB1 was accomplished using two different CTNNB1-specific siRNAs, which when tested in a functional assay using a Beta-catenin-Activated Reporter (BAR) [28] were able to suppress BAR activity by ∼99% and ∼97%, respectively. We performed Gene set enrichment analysis (GSEA) using the modified Kolmogorov–Smirnov (KS) test [29] to evaluate the CTNNB1-bound genes for their distribution in the microarray dataset wherein the genes were rank-ordered for their differential expression between control and CTNNB1-depleted cells. The KS test revealed a highly significant correlation (*p*  =  0) between CTNNB1 binding and gene expression changes after CTNNB1 knockdown, and this was true with both siRNAs (Figure 3A and data not shown). A majority of the CTNNB1-bound genes were positively correlated, whereas a small number of the genes were negatively correlated, with CTNNB1 expression (Figure 3B). This is consistent with CTNNB1 mainly acting as a transcriptional activator but also being capable of repressing the expression of certain genes [30].

**Figure 4:**
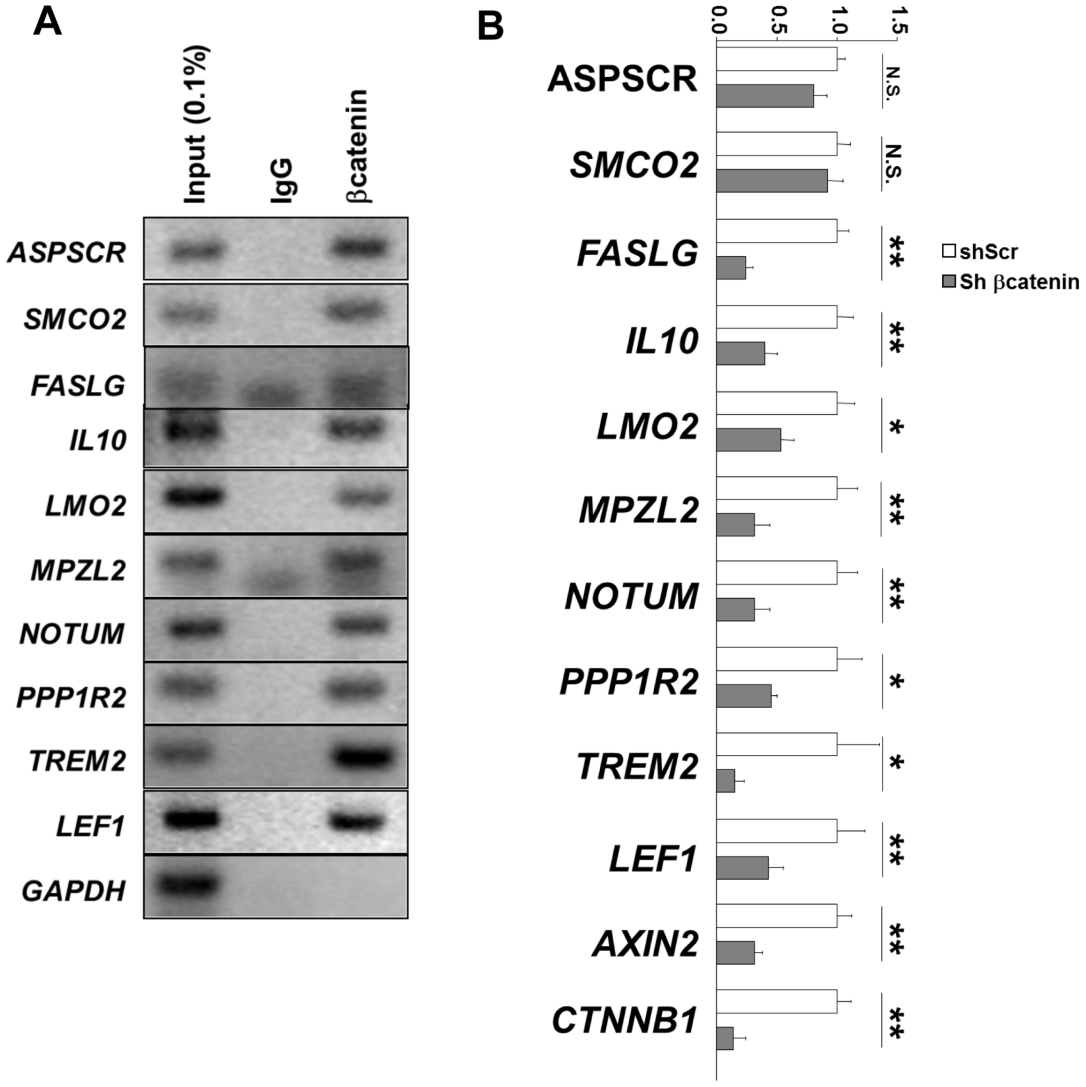
Validation of CTNNB1 direct target genes. *A*. ChIP-PCR analysis of the indicated genes using an independently prepared chromatin sample. *LEF1* and *GAPDH* serve as positive and negative controls, respectively. *B*. Quantitative RT-PCR of the indicated genes. Mean ± standard deviation of 4 independent experiments are shown. *p* values are calculated with two-tailed standard *t-*test. N.S.: not significant, *: *p*<0.05, and **: *p*<0.01.

Using a combinatorial criteria, i.e., down-regulation in microarray analysis with *p*<0.01 and log ratio <–0.2 following treatment of both siRNAs and displaying CTNNB1 binding peaks within ±20 kb from the TSS, we identified 162 genes as direct and functional Wnt targets (Table S1). Included in this target signature are known targets such as *AXIN2, CDX2, ID2, LEF1, LGR6, MSX1, NKD1, NKD2, SP5, TDGF1,* and *TNFRSF19*, indicating the validity of the signature. We also sampled 10 genes from the list, and used ChIP-PCR to confirm CTNNB1 binding at the expected sites (Figure 4A). Using an shRNA against CTNNB1 that is different from the two siRNAs used in the microarray analysis, we validated that knockdown of CTNNB1 resulted in a significant down-regulation of most of the tested genes including *FASLG, IL10, LMO2, MPZL2, NOTUM, PPP1R2,* and *TREM2* (Figure 4B).

Gene Ontology (GO) term enrichment by Database for Annotation, Visualization, and Integrated Discovery (DAVID) [31] of the 162-target-signature suggested important developmental functions of the Wnt pathway, including embryonic appendage morphogenesis, heart, or muscle development (Table S2). GO analysis also revealed genes involved in several signaling pathways, suggestive of potential cross-talks between Wnt signaling and these pathways. Specifically, significantly detected pathway GO terms include cytokine signaling (*FASL, BMP7, CXCL4, CXCL6, CXCL14, EDAR, IL10RA, IL10, TNFRSF1B, TNFRSF11B, TNFRSF19*), pathways in cancer (*FASL, GLI3, AXIN2, FGF3, FGF9, FGF19, LEF1, WNT6, WNT11*), or Hedgehog signaling pathway (*GLI3, BMP7, WNT6, WNT11*) (Table 2).

**Table 2:** Summary of DAVID GO analysis (pathways) showing top categories in the 162 CTNNB1 target gene set.

Category	Ontology term	*p*-value
KEGG pathway	Cytokine-cytokine receptor interaction	5.8e-5
KEGG pathway	Basal cell carcinoma	1.2e-3
KEGG pathway	Pathways in cancer	6.3e-3
KEGG pathway	Wnt signaling pathway	9.2e-3
KEGG pathway	Hedgehog signaling pathway	1.2e-2
Panther pathway	P00044: Nicotinic acetylcholine receptor signaling pathway	1.9e-2
KEGG pathway	T cell receptor signaling pathway	6.6e-2
Panther pathway	P00057: Wnt signaling pathway	9.1e-2

GO analysis also identified genes encoding Wnt signaling components as CTNNB1 targets and we generated an extended list of potential feedback regulators (Table 3). *AXIN2, LEF1, NKD1* and *NKD2* have been described as negative or positive feedback regulators of the pathway [25], [32], [33]. *WNT6* and *WNT11* encode Wnt ligands that have potential tumor-promoting roles even in CRCs with *APC* mutations [34] and that have the capability to signal to the stromal components to modulate the tumor microenvironment [35]. Other potential feedback regulators include *APCDD1*, *NOTUM, PPP1R2*, and *ZNRF3*. *APCDD1* is reported to negatively regulate the pathway at the ligand-receptor interaction step and its mutation is responsible for an autosomal dominant hair loss disease, hereditary hypotrichosis simplex [36]. *NOTUM* encodes a secreted α/β-hydrolase, which is known to antagonize Wnts by releasing glypicans from the cell surface [37]. *PPP1R2* gene product inhibits the protein phosphatase 1 complex, which regulates the phospholyration status of GSK3 [38] and consequently CTNNB1 degradation [39]. *ZNRF3* encodes an E3 ubiquitin ligase that specifically promotes ubiquitination and degradation of Frizzled receptors [40], [41]. As shown above, we have confirmed *NOTUM* and *PPP1R2* to be bona fide targets of CTNNB1. Taken together, these data suggest that Wnt signaling is subject to complex feedback regulations.

**Table 3:** Summary of feedback regulators of the Wnt signaling pathway that are part of the 162-target-signature.

Gene symbol	Description	Function in Wnt pathway	References
*APCDD1*	Adenomatosis polyposis coli down-regulated 1	Negatively regulate the pathway at the ligand-receptor interaction.	[36]
*AXIN2*	Axin 2 (conductin, axil)	Promote CTNNB1 degradation.	[25]
*LEF1*	Lymphoid enhancer-binding factor 1	Mediate CTNNB1-induced transcription. Dominant negative forms exist.	[32]
*NKD1*	Naked cuticle homolog 1 (Drosophila)	Negatively regulate the pathway by acting between Disheveled-CTNNB1	[33]
*NKD2*	Naked cuticle homolog 2 (Drosophila)	Negatively regulate the pathway by acting between Disheveled-CTNNB1	[33]
*NOTUM*	Notum pectinacetylesterase homolog (Drosophila)	Antagonize the pathway by releasing glypicans from cell surface.	[37]
*PPP1R2*	Protein phosphatase 1, regulatory (inhibitor) subunit 2	Increase GSK3 phosphorylation. (potential role in Wnt pathway)	[39]
*WNT11*	Wingless-type MMTV integration site family, member 11	Wnt ligand.	[52]
*WNT6*	Wingless-type MMTV integration site family, member 6	Wnt ligand.	[52]
*ZNRF3*	Zinc and ring finger 3	Transmembrane E3 Ubiqutin ligase. Promote turnover of Wnt receptors/co-receptors.	[40], [41]

### The CTNNB1 target signature is enriched in intestinal stem cells and CRCs, but does not correlate with the survival of CRC patients

**Figure 5:**
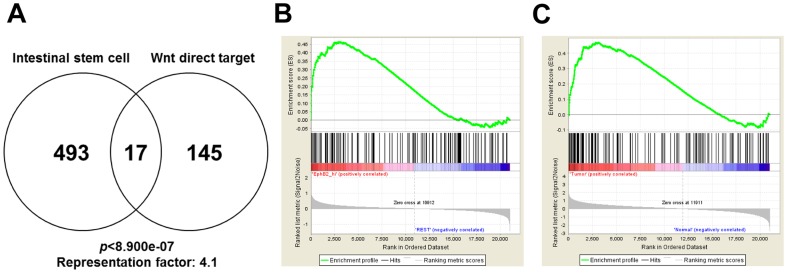
Enrichment of the 162-gene CTNNB1 target in intestinal stem cells and CRC samples. *A*. Overlap between the CTNNB1 target gene set and the Lgr5^+^ intestinal stem cell gene set. *p* value and representation factor highlight statistical significance as compared to random chance. Calculation was based on the assumption that the number of total genes is 20000. *B-C*. GSEA for the 162 genes using comparative data between EphB2^high^ human intestinal stem cell and EphB2^low^ nonstem cell populations (*B*), or between colon cancer and normal tissue samples (*C*).

Lgr5^+^ intestinal cells are actively cycling stem cells and the Wnt/CTNNB1 signaling pathway plays a critical role in their self-renewal [2]. We therefore compared the CTNNB1 target signature identified in this study with a recently reported gene signature (a set of 510 genes identified from microarray-based transcriptional profiling and mass spectrometric protein profiling) that is enriched in Lgr5^+^ intestinal stem cells [42]. A statistically significant overlap (*p*<8.9e-07, representation factor > 4.1; comparing to random chance) was found: 17 genes (∼10%) in the CTNNB1 target gene signature were also part of the Lgr5^+^ intestinal stem cell signature (Figure 5A, Table 4). GSEA on a microarray data set from an EphB2-enriched intestinal stem cell population of human colon epithelial cells (GSE31257) [43] also revealed the enrichment of our 162-target-signature in these cells [Normalized Enrichment Score (NES)  =  1.81, FDR *q*-value  =  0.0; Figure 5B]. These data suggest that the direct CTNNB1 target genes in SW480 cells are also activated in normal intestinal stem cells.

**Table 4:** Summary of the 17 common genes in CTNNB1 target signature and Lgr5^+^ intestinal stem cell signature.

Gene symbol	Description	Fold change shRNA_1	Fold change shRNA_2	*p* value shRNA_1	*p* value shRNA_2
*SP5*	Sp5 transcription factor	–25.11886432	–17.7828	2.65E-35	3.04E-38
*TNFRSF19*	Tumor necrosis factor receptor superfamily, member 19	–20.89296131	–13.1826	0	1.16E-33
*AXIN2*	Axin 2 (conductin, axil)	–13.18256739	–9.33254	0	0
*KRT23*	Keratin 23 (histone deacetylase inducible)	–11.22018454	–8.31764	1.54E-35	7.37E-29
*APCDD1*	Adenomatosis polyposis coli down-regulated 1	–6.760829754	–4.7863	1.69E-12	5.43E-10
*HUNK*	Hormonally upregulated Neu-associated kinase	–5.370317964	–4.2658	2.09E-21	1.18E-15
*RASL11B*	RAS-like, family 11, member B	–5.011872336	–5.49541	5.68E-20	1.12E-22
*NELF*	Nasal embryonic LHRH factor	–4.265795188	–4.2658	3.17E-20	2.38E-25
*IL17RD*	Interleukin 17 receptor D	–3.981071706	–3.16228	1.35E-07	1.75E-12
*ZNRF3*	Zinc and ring finger 3	–3.89045145	–3.31131	9.75E-19	1.61E-19
*DOCK11*	Dedicator of cytokinesis 11	–3.630780548	–4.36516	2.22E-17	5.23E-15
*SORBS2*	Sorbin and SH3 domain containing 2	–3.467368505	–3.16228	3.29E-07	2.02E-05
*PKIG*	Protein kinase (cAMP-dependent, catalytic) inhibitor gamma	–3.311311215	–3.46737	5.92E-40	1.61E-42
*LRIG1*	Leucine-rich repeats and immunoglobulin-like domains 1	–2.570395783	–1.77828	2.81E-15	2.81E-07
*EFNA4*	Ephrin-A4	–2.344228815	–1.77828	7.14E-12	9.15E-07
*FRAS1*	Fraser syndrome 1	–2.290867653	–2.39883	1.99E-10	2.27E-08
*ACSS2*	Acyl-CoA synthetase short-chain family member 2	–1.905460718	–1.8197	1.11E-11	1.19E-08

**Figure 6:**
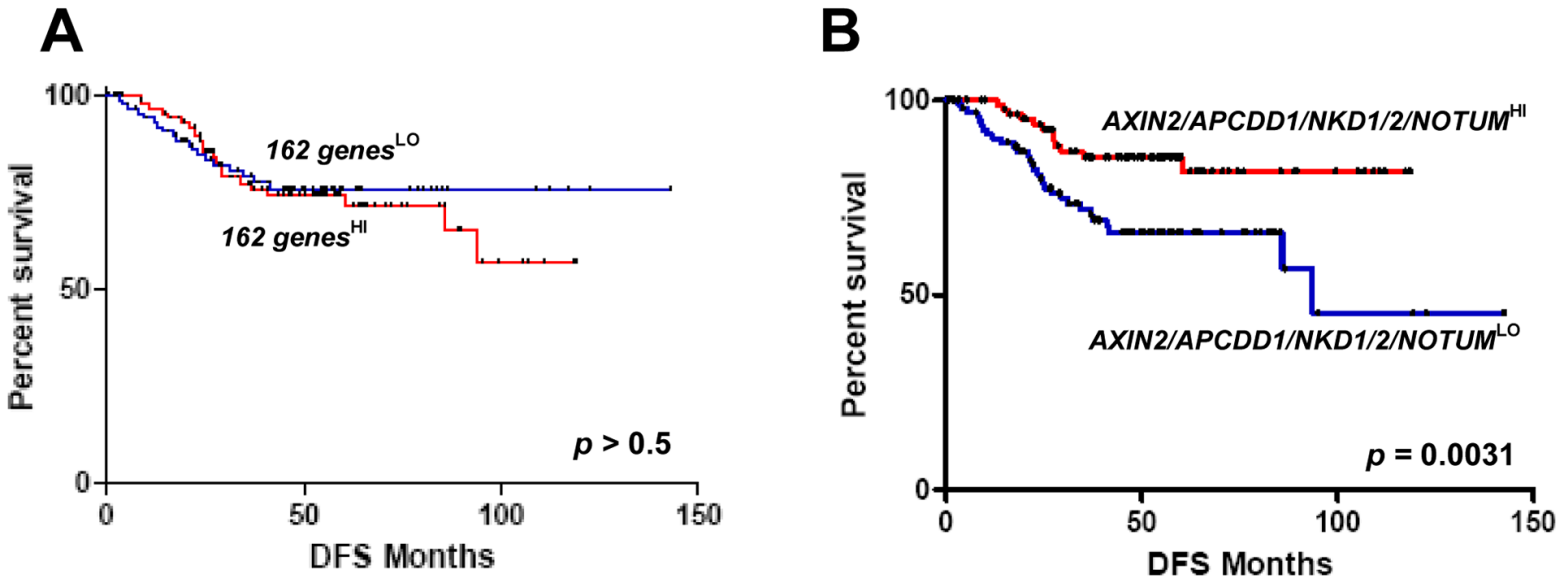
Kaplan-Meier curves for disease-free survival (DFS) of colon cancer patients with the indicated gene expression patterns. A total of 232 patients were categorized into high (red) or low expressers (blue) based on the expression of the 162 genes (*A*) or five negative feedback genes (*AXIN2, APCDD1, NKD1, NKD2, NOTUM)* (*B*). *p* values were calculated by log rank analysis.

To probe the relevance of the 162-target-signature in clinical specimens, we performed GSEA on gene expression data from human colon cancer samples (GSE41328) [44]. This analysis revealed a striking enrichment of the CTNNB1 target signature in colon cancer samples compared to normal colon tissues (NES  =  2.01, FDR *q*-value  =  0.0; Figure 5C). We also used a publically available gene expression data set to test whether the 162-target-signature can predict the outcome of colon cancer patients [45]. Despite the enrichment of the signature in colon cancer samples, we did not observe any significant correlation between the overall expression of the signature and disease-free post-surgery survival of colon cancer patients (Figure 6A). This said, the expression of several of the negative feedback regulators that we identified in this work correlated with a better disease-free survival rate. For example, patients with high average expression of *AXIN2, APCDD1, NKD1, NKD2*, and *NOTUM* showed significantly longer disease-free survival compared to the low expresser group (Figure 6B). Together, our results reveal an enrichment of direct CTNNB1 target genes in intestinal stem cells and clinical CRC samples. However, a significant correlation with prognosis of CRC patients is limited to a select subset of CTNNB1 target genes, namely the negative feedback factors, rather than the entire target signature.

## Discussion

Our unbiased genome-scale screening for CTNNB1 target genes combining ChIP-seq and microarray analyses provides an important resource for colon cancer research. Despite the significant correlation between chromatin binding and gene expression regulation, our work uncovers a small number (162) of genes as direct, functional CTNNB1 targets in SW480 colon cancer cells. This number is considerably smaller than that of CTNNB1-bound loci or of CTNNB1-responsive genes, suggesting that not all bound loci are functionally utilized in SW480 cells for transcriptional regulation, and that much of the changes in gene expression occurs as indirect consequences of CTNNB1 depletion. We might have missed some modestly regulated targets by requiring the gene expression change to occur upon treatment with two different siRNAs and by using stringent cut-off values. Some of the CTNNB1-bound loci may be regulated in a context-specific manner; for example, genes that have additional layers of regulation such as those silenced by DNA methylation or predominantly controlled by other transcriptional/translational regulators in SW480 cells may not show a significant decrease in transcription upon CTNNB1 depletion. It remains possible that such loci are regulated by CTNNB1 in other cellular contexts.

Our validation experiments suggest an 80% success rate in the identification of bona fide CTNNB1 targets using the genomic approach. A difference in knockdown efficiency among si/shRNAs or off-target effects of si/shRNAs may account for the “false positives”. The CTNNB1 target signature of 162 genes presents some interesting features. Most striking is the presence of feedback regulators of the pathway. Feedback regulation of the Wnt/CTNNB1 pathway has been well-established based on studies of individual genes [25]. Using serial analysis of chromatin occupancy (SACO), CTNNB1 occupancy of components of the canonical Wnt signaling pathway has been discovered [11]. Our finding that Wnt pathway components are overrepresented in the CTNNB1 target signature adds additional evidence to support this important mode of regulation. Of note, our study and the SACO study [11] uncover largely distinct Wnt pathway genes as CTNNB1 targets: out of the 15 genes that were identified in the SACO study, only 2 genes (*AXIN2* and *WNT11*) were found in our list of 10 Wnt pathway genes (Table 2). Furthermore, when compared with another CTTNB1 ChIP-seq study that used HCT116 cells [12], an overlap of 161 CTNNB1-bound genes was found between the 988 genes identified in that study and the 2794 genes identified in current study. Even though the overlap is statistically significant (*p*<4.8e-07), a substantial number of target genes was specific to each study. This may be in part due to a difference in parameter setting or potential noises in the analysis, but could also reflect a real difference in CTNNB1 function between the CRC cell lines (HCT116 vs. SW480) used in the two studies. Indeed, a difference in the DNA methylation patterns of Wnt target genes between HCT116 and SW620, a lymph node metastatic variant of SW480, has been reported [46]. As such, our work reveals new facets of feedback regulation of Wnt/CTNNB1 signaling. Fine-tuning the Wnt signaling output by multiple feedback mechanisms may be important for the precise spatiotemporal regulation of its activity during tissue patterning and maintenance of tissue homeostasis. In addition, malfunction of these mechanisms may contribute to pathological conditions such as cancer [47].

Even for a relatively small number of genes in the 162-target-signature, the related functional terms appear undefined and diverse. This is consistent with the divergent biological effects of Wnt/CTNNB1 signaling [2]. The signature includes a number of transcriptional regulators (Table S1), implicating the existence of secondary/indirect transcriptional targets. The identification of direct CTNNB1/TCF targets by others [11]–[14] and this work provides a framework to dissect the relative contributions of direct and indirect target genes to the biological effects of the pathway.

It is widely recognized that deregulation of the Wnt/CTNNB1 pathway is a key initiation step of intestinal tumorigenesis [48]. This deregulation is thought to occur in the intestinal stem cell population that fuels tumor growth [5], [6]. Consistent with this notion, we found the CTNNB1 target signature to be significantly enriched in isolated intestinal stem cells as well as in clinical CRC samples. However, we did not observe a significant correlation between the expression of the target signature and the prognosis of CRC patients. This is interesting considering that elevated expression of an intestinal stem cell signature predicts poor prognosis of CRC patients [46], [49] and that deregulation of Wnt pathway has been known as a hallmark of CRC [50]. The fact that the CTNNB1 target signature includes a number of feedback factors may complicate the scenario. For example, suppression of the negative feedback regulators might turn on the oncogenic Wnt signaling activity in cancer cells, but since these feedback factors themselves are Wnt targets, the overall Wnt target gene expression pattern may not seem to correlate with prognosis. Indeed, silencing of Wnt target genes by promoter methylation has been noted in CRCs with poor prognosis [46]. Our results show that the expression of select negative feedback factors from our gene list indeed correlates positively with the survival of CRC patients (Figure 6B). In such cases where the negative feedback regulators of the pathway are silenced, the average target gene expression may not reflect the actual signaling activity of the pathway. Thus, the current study and others [46] suggest that careful considerations of the complex regulations and context dependence are warranted regarding the clinical utility of various Wnt target gene sets that have emerged in the literature. Our study adds to a comprehensive view of Wnt signaling regulation in CRCs, which has been studied for decades but yet to be fully understood.

## Conclusion

Our data provide a genome-wide view of gene regulation by Wnt/CTNNB1 signaling in CRCs. The target gene analysis revealed multiple layers of feedback regulation of the Wnt/CTNNB1 pathway. The CTNNB1 direct target gene signature was highly expressed in intestinal stem cell populations and cancer cells, but did not show significant correlation with the survival of CRC patients. This study provides an important resource to understand the complex and divergent functions of Wnt/CTNNB1 pathway.

## Methods

### Cell culture

SW480 cells were obtained from ATCC and maintained in Dulbecco’s modified eagle medium supplemented with 10% fetal bovine serum.

### Chromatin immunoprecipitation (ChIP) and high-throughput sequencing

ChIP was performed according to the protocol from Millipore with some modifications. SW480 cells were cross-linked first with a cocktail of protein-protein crosslinkers [0.67 mM disuccinimidyl sulfate (DSS), 0.67 mM disuccinimidyl glutarate (DSG), and 0.67 mM ethylene glycolbis (succinimidylsuccinate) (EGS)] [20] for 45 minutes at room temperature, and then in 0.75% formaldehyde for 10 minutes at 37°C. After washing, chromatin was sheared into ∼100–300-bp fragments using a Bioruptor (Diagenode Inc.). Lysates were precleared with Dynabeads protein A (Invitrogen) for one hour at 4°C, and small aliquots of the recovered supernatant were subjected to reverse crosslinking and purification of DNA (as input samples). Input samples were used to measure the concentration of chromatin DNA and immunoprecipitation was performed with 25 μg of DNA-containing chromatin at 4°C overnight with control IgG (Santa Cruz Biotechnology, sc-2027) or anti-CTNNB1 antibody (Santa Cruz Biotechnology, sc-7199). Immuno-complexes were then purified with Dynabeads protein A and reverse crosslinked by incubating with 200 mM NaCl at 65°C for 5 hours. After proteinase K treatment for 1 hour, ChIP DNA was recovered using the QIAquick PCR purification kit (Qiagen, 28104). Library generation was performed using pooled ChIP DNA samples from four independent ChIP preparations using the Illumina protocol. Briefly, ChIP DNA fragment ends were repaired and phosphorylated using Klenow, T4 DNA polymerase and T4 polynucleotide kinase (Illumina kit components). After ligation of Illumina adapters, DNA was size selected by gel purification and then PCR amplified using Illumina primers. Sequencing was performed at Ambry Genetics on an Illumina Genome Analyzer IIx, one lane per sample, 36-bp singleton sequencing.

The validation experiment was performed using ChIP-PCR with primers listed in Table S3.

### Microarray analysis

The analysis was published previously [27], and experimental details are as follows. Two-color microarray was performed in duplicates for SW480 cells transfected with control (siRNA against luciferase gene) and two independent siRNAs against CTNNB1 (CTNNB1#1 forward; CUAUCUGUCUGCUCUAGUATT, reverse; UACUAGAGCAGACAGAUAGTT, CTNNB1#2 forward; CUGUUGGAUUGAUUCGAAATT, reverse; UUUCGAAUCAAUCCAACAGTT), respectively. Twenty-four hours after transfection, RNA was extracted from the cells using the RNeasy kit (Qiagen, Valencia, CA). cDNA was synthesized with Cy3 (control) and Cy5 (CTNNB1 siRNA) labelling, using a custom automated version of the aminoallyl MessageAmp II kit (Life Technologies). Labelled samples were hybridized with Rosetta/Merck Human 44k 1.1 microarray (GPL4372) by incubating at 40°C for 48 hours in a rotating carousel. After washing to remove non-specific hybridized samples, microarrays were dried in an ozone-free nitrogen chamber. Microarrays were scanned using the Agilent LP2 laser scanner. The scanner output is a Tiff file, which contains quantitative hybridization data from each individual microarray. The Tiff files were then processed using Rosetta custom feature extraction software, which performs error modeling. Data were then processed using the Rosetta Resolver® system, which performs a squeeze operation that combines replicates of the same sequence in an array while applying error weighting. The error weighting consisted of adjusting for additive and multiplicative noise. A *p*-value was generated and propagated throughout the system.

### Bioinformatic analysis of ChIP-seq and microarray data

After Illumina sequencing, reads were mapped to a reference genome by Ambry Genetics using ELAND, allowing one mismatch. Short sequence reads that mapped to simple and complex repeats or that were not unique by chance were removed from the analysis. BED files were created and used as input for downstream data processing, as well as for visualization in the UCSC Genome Browser (http://genome.ucsc.edu/index.html). Peak identification was accomplished using MACS [21] with the cutoff parameters of *mfold* 10, *bandwidth* 300 bp, *p*-value 10^−5^ and false discovery rate (FDR) of 5%. For each peak, the distance from the peak to the nearest TSS was determined, and plotted. The TSSs were taken from a RefSeq file obtained from NCBI. The background was determined by placing peaks at random locations on the genome and by determining distances to TSS. For motif analysis, DNA sequences were retrieved using Galaxy (http://main.g2.bx.psu.edu) and used for motif search using MEME [22] with 1500 randomly selected 5% FDR peaks with an average fragment size of 320.6 bp. KS analysis, a modified method of GSEA [29], was used to compare ChIP-seq data with microarray data. A KS plot was obtained by calculating the running sum statistics for the ChIP-seq gene set to observe enrichment in the ranked gene list from expression microarray data.

The ChIP-seq and microarray data have been deposited into the Gene Expression Omnibus (GEO) database. The accession numbers are GSE 53927 and GSE53656, respectively.

### Reverse transcription and quantitative PCR

Total RNA was extracted using the RNeast kit (Qiagen, Valenc) and cDNA was prepared from 1 μg total RNA using Retroscript High Capacity cDNA Reverse Transcription kit (Life Technologies) according to manufacturer’s instructions. Quantitative PCR was performed using a CFX96 qPCR system and SsoAdvanced SYBR Green supermix (Bio-rad). House keeping gene GAPDH was used for normalization. Primers used for gene expression analysis are listed in Table S3.

### Survival analysis

A cohort of 232 unique CRC expression profiles with clinical outcome data was obtained from GSE17538 [45]. This is a metacohort composed of 177 CRC patients treated at L. Moffitt Cancer Center (Tampa, USA) and 55 CRC patients treated at Vanderbilt University Medical Center (Vanderbilt, USA). Each array was log2 normalized and mean centered. Expression values for the 162 Wnt target genes were extracted from each array and mean expression of these genes was calculated for each array. Patients were divided into high and low expressing groups using the median of these values. Kaplan-Meier survival curves were generated for each group and the log-rank test was used to compare the two curves.

### Availability of supporting data

The data sets supporting the results of this article are included in the article and its supplemental files.

## Supporting Information

Table S1 **List of direct and functional β-catenin targets.**
(XLS)

Table S2 **GO terms enriched in DAVID analysis.**
(XLS)

Table S3 **RT-qPCR/ChIP-PCR primers used in this study.**
(DOC)
