# Supplementary material for: Integrative ChIP-seq/Microarray Analysis Identifies a CTNNB1 Target Signature Enriched in Intestinal Stem Cells and Colon Cancer
Source: PLoS One. 2014 Mar 20;9(3):e92317. doi: 10.1371/journal.pone.0092317 (PMC3961325; doi:10.1371/journal.pone.0092317)
Supplement: Table S3 — RT-qPCR/ChIP-PCR primers used in this study. (DOC) [file pone.0092317.s003.doc]

**Table S3. RT-qPCR/ChIP-PCR primers used in this study**

| **Name** | **Sequence** | **Description** |
| --- | --- | --- |
| RT-qPCR primers | | |
| *ASPSCR* | CCTGAGAACATGGTTCGCATC | forward |
| *ASPSCR* | GGCCTGAACAGAAAGAGTCCT | reverse |
| *SMCO2* | ATGTGACTGAAGGTGCAATGC | forward |
| *SMCO2* | ATCTGTCTAAGATGTGGTCCACT | reverse |
| *FASL* | TGCCTTGGTAGGATTGGGC | forward |
| *FASL* | GCTGGTAGACTCTCGGAGTTC | reverse |
| *IL10* | TAGAATGGGAGGCAGGTTTG | forward |
| *IL10* | GGGGCCTGAAGCTCTCTACT | reverse |
| *LMO2* | GCAGAAGGAGACCAGAGGTG | forward |
| *LMO2* | TAAGACGGGAAAGAGCCAGA | reverse |
| *MPZL2* | GGAATCCTGAGCGGTACGATG | forward |
| *MPZL2* | CTGGCAGGTGTATGTCCCATT | reverse |
| *NOTUM* | CTTCATGGCGCAAGTCAAGAG | forward |
| *NOTUM* | CGAGGTGTTGAGTAGGAGGTG | reverse |
| *PPP1R2* | AACAAGACCTCTACGACTTCCT | forward |
| *PPP1R2* | TGATACGTCGCCAAGATGTTC | reverse |
| *TREM2* | AGCCATCACAGACGATACCC | forward |
| *TREM2* | CTGGTAGAGACCCGCATCAT | reverse |
| *LEF1* | ATGTCAACTCCAAACAAGGCA | forward |
| *LEF1* | CCCGGAGACAAGGGATAAAAAGT | reverse |
| *AXIN2* | GTGATGGAGGAAAATGCCTACC | forward |
| *AXIN2* | GTCCCCCATTACTCATGTAAGC | reverse |
| *CTNNB1* | TTCGAAATCTTGCCCTTTGTCCCG | forward |
| *CTNNB1* | AATTCGGTTGTGAACATCCCGAGC | reverse |
| *GAPDH* | GGACCTGACCTGCCGTCTAGAA | forward |
| *GAPDH* | GGTGTCGCTGTTGAAGTCAGAG | reverse |
| ChIP-PCR primers | | |
| *ASPSCR* | GTTTGATGTCACGCTGCTGT | forward |
| *ASPSCR* | GAGATGGTCATGAGGCCACT | reverse |
| *SMCO2* | ACACCCTCAGGAAATGGACA | forward |
| *SMCO2* | GTGATGGCACCATCCTTTTT | reverse |
| *FASL* | TGCTGAAATTCCCACCCTTA | forward |
| *FASL* | CCAGGCTCTCACACATCAAA | reverse |
| *IL10* | TAGAATGGGAGGCAGGTTTG | forward |
| *IL10* | GGGGCCTGAAGCTCTCTACT | reverse |
| *LMO2* | GCAAATCCAAAAGTGGGAAA | forward |
| *LMO2* | TCCTGGGTCCAGCAATAAAG | reverse |
| *MPZL2* | AGGCAGTGTTGTGGTCTTCC | forward |
| *MPZL2* | GAATGGATCTGGGCACTGAG | reverse |
| *NOTUM* | GAGGCAGAAACAAACCCTCA | forward |
| *NOTUM* | TAGACAGCTGCTCCCAGGAT | reverse |
| *PPP1R2* | GAGTCCAGACAGAGCCCATT | forward |
| *PPP1R2* | CGGCATCTAGCAAGATCAAA | reverse |
| *TREM2* | CCAAAGGGACATCAAAAGGA | forward |
| *TREM2* | GGAGCCATCAGTAGGAGCTG | reverse |
| *LEF1* | TCCTGGATTCCTTCACCAAC | forward |
| *LEF1* | TCAGGCTGCTGAACATTGAA | reverse |
| *GAPDH* | CTGAGCAGACCGGTGTCACATC | forward |
| *GAPDH* | GAGGACTTTGGGAACGACTGAG | reverse |
